# Micro/Nano Multilayered Scaffolds of PLGA and Collagen by Alternately Electrospinning for Bone Tissue Engineering

**DOI:** 10.1186/s11671-016-1532-4

**Published:** 2016-07-04

**Authors:** Sanghwa Kwak, Adnan Haider, Kailash Chandra Gupta, Sukyoung Kim, Inn-Kyu Kang

**Affiliations:** Department of Polymer Science and Engineering, Kyungpook National University, 80, Daehak-ro, Buk-gu, Daegu, 702-701 South Korea; Polymer Research Laboratory, Department of Chemistry, I.I.T. Roorkee, Roorkee, 247667 India; School of Materials Science and Engineering, Yeungnam University, Gyeongbuk, 712-749 South Korea

**Keywords:** Micro/nano mixed fibrous scaffolds, PLGA, Collagen, Hydroxyapatite, Dual extrusion electrospinning

## Abstract

The dual extrusion electrospinning technique was used to fabricate multilayered 3D scaffolds by stacking microfibrous meshes of poly(lactic acid-co-glycolic acid) (PLGA) in alternate fashion to micro/nano mixed fibrous meshes of PLGA and collagen. To fabricate the multilayered scaffold, 35 wt% solution of PLGA in THF-DMF binary solvent (3:1) and 5 wt% solution of collagen in hexafluoroisopropanol (HFIP) with and without hydroxyapatite nanorods (nHA) were used. The dual and individual electrospinning of PLGA and collagen were carried out at flow rates of 1.0 and 0.5 mL/h, respectively, at an applied voltage of 20 kV. The density of collagen fibers in multilayered scaffolds has controlled the adhesion, proliferation, and osteogenic differentiation of MC3T3-E1 cells. The homogeneous dispersion of glutamic acid-modified hydroxyapatite nanorods (nHA-GA) in collagen solution has improved the osteogenic properties of fabricated multilayered scaffolds. The fabricated multilayered scaffolds were characterized using FT-IR, X-ray photoelectron spectroscopy, and transmission electron microscopy (TEM). The scanning electron microscopy (FE-SEM) was used to evaluate the adhesion and spreads of MC3T3-E1 cells on multilayered scaffolds. The activity of MC3T3-E1 cells on the multilayered scaffolds was evaluated by applying MTT, alkaline phosphatase, Alizarin Red, von Kossa, and cytoskeleton F-actin assaying protocols. The micro/nano fibrous PLGA-Col-HA scaffolds were found to be highly bioactive in comparison to pristine microfibrous PLGA and micro/nano mixed fibrous PLGA and Col scaffolds.

## Background

In tissue engineering, the structures and properties of scaffolds play a significant role in controlling the activity of the seeded cells. The scaffolds need to be compatible with neighboring tissues and possess attractive sites for cells adhesion. To fabricate bioactive scaffolds, various methods, such as phase separation [1], gas foaming [2], porogen leaching [3], emulsion freeze-drying [4], and solid free-form fabrications [5], have been used frequently, but out of these methods of fabrication of scaffolds, the technique of electrospinning is found to be more acceptable. The electrospinning is found to be a versatile and simple technique in the fabrication of bioactive fibrous scaffolds of different sizes [6] using various biodegradable materials [7–9]. The electrospinning parameters [10], such as tip to collector distance, field strength of grounded electrode, and solution viscosity, have played a prominent role in controlling the properties of the scaffolds. During electrospinning process, a strong field is applied to elongate the drop of polymer solution held by the surface tension at the tip of the capillary. As a result of it, a solution cone is formed due to the coupling action of electrostatic repulsion within the charged droplet of polymer solution and attraction force applied through a grounded electrode of the opposite polarity. On further increasing the electrode field intensity, the formation of fiber takes place as the solution surface tension is overcome by the applied field strength. However, the overall properties of the fibers depend on various parameters, which need to be optimized to obtain fibers of desired morphology, microstructures, and their diameter. Amongst the various parameters, the viscosity of the polymer solution is found to be highly important in controlling the morphological structures and thickness of fibers; hence, solvent properties [11, 12] and humidity have played a significant role [13] in the fabrication of scaffolds by electrospinning process. The process of electrospinning is efficient in forming continuous and uniform fibers from micro- to nano-sized diameter [14–16] for various applications ranging from cell seeding to the delivery of drugs and genes as regenerative medicines [17–21]. There is a great challenge to design a suitable scaffold to elicit the specific response of local cells or organs to develop tissues or organs of desired functionality [22, 23]. In comparison to other scaffolds, the nanofibrous non-woven scaffolds have shown enhanced bioactivity due to high surface-area-to-mass ratio and 3D nanostructures [24, 25]. The nanofibrous non-woven scaffolds are able to control cell adhesion, proliferation, and differentiation in tissue engineering. The scaffolds that mimic the supramolecular and biological function of extracellular matrices (ECM) is a key issue in designing the artificial scaffolds for tissue engineering and development of artificial organs [26–28]. The scaffolds in various designs and architectures have been fabricated by the technique of electrospinning to facilitate the organization and differentiation of the cells to a new tissue with improved performances. The electrospinning technique is used to fabricate cytocompatible core shell nanocomposite scaffolds for enhanced drug loading and cell adhesion in tissue engineering [29–31]. The mixed fiber mesh scaffolds have been fabricated using a special technique of periodically transverse movable collector [32], which provides an opportunity to develop scaffolds with enhanced properties in comparison to properties of individual polymer scaffolds. Amongst the various biomaterials, the biodegradable PLGA has shown a great potential as a carrier for drug delivery and in fabrication of scaffolds for tissue engineering [33–35]. Recently, considerable efforts have been made to develop suitable scaffolds for tissue engineering using various biodegradable polymers, such as collagen and polymer/collagen blends. Amongst the various types of collagen, the collagen type I is a main structural component of natural extracellular matrices [36] and comprises about 70–80 wt% of natural tissues. The collagen consisted of elongated fibers forming rod-like triple helices, which are stabilized by intramolecular hydrogen bonding [37]. It forms self-assembled biocompatible and insoluble fibrils of high mechanical strength with low immunogenicity [38]; hence, collagen becomes a natural choice for biomedical and tissue engineering. It helps in attachment, cellular penetration, and wound repair. Various bioactive ceramics, such as calcium phosphates, silica, alumina, zirconia, and titanium dioxide, are found to be useful in bone tissue regeneration due to their osteoinductive properties [34, 39, 40]. The osteoinductivity of the silica is considered due to its bonding ability directly to the soft and hard tissues by producing HA through silanol interactions with calcium and phosphate ions of biological fluids [41]. Calcium phosphate and calcium hydroxide are used frequently in the field of dentistry, orthopedics, and plastic surgery [42–44] but due to their slow degradability, not found suitable in comparison to osteoconductive HA and its derivatives [45–47]. The poor processibility and mechanical strength of these ceramics including silica have decreased their suitability in the fabrication of the scaffolds; hence, these bioceramics are blended with various synthetic biomaterials, such as poly(lactic acid), poly(glycolic acid), poly(lactic-co-glycolic acid), and poly(ε-caprolactone), for the fabrication of scaffolds for tissue engineering [48, 49]. Due to the lack of cell recognition sites on synthetic polymers, the blending of collagen with synthetic polymers is found to be useful [50]. However, the main problem with collagen is its antigenicity and the difficulties in its processing [51]. The coating of collagen-hydroxyapatite composite on PLGA/β-tricalciumphosphate (β-TCP) skeleton has shown a significant improvement in alkaline phosphatase activity, which indicated that collagen-hydroxyapatite composite, has played a significant role in controlling the bioactivity of PLGA/β-TCP-based scaffolds [52]. These studies have clearly indicated that the combination of collagen and hydroxyapatite is useful to provide favorable environment to control the biological activity of scaffolds. To achieve stable dispersion of hydroxyapatite in collagen and other biomaterials for the fabrication of the scaffold, the modification of hydroxyapatite with hydrophilic materials such as succinic acid has been carried out successfully in previous study [33]. However, other approaches, such as hydrolysis by alkali treatment [53], plasma treatment [54], and ion irradiation techniques [55], have also been tried. But chemical modification of HA with succinic acid is found to be more convenient and useful for grafting of insulin and its release applications [34]. In comparison to silica and other ceramics, the HA is also osteogenic but its bioactivity largely depends on its available surface area. The HA has been used either as nanoparticles or as 1D nanostructures, such as nanotubes/nanorod or nanowires, in the fabrication of nanofibrous scaffolds for tissue engineering. The 1D nHA is found to be more osteogenic in comparison to HA in other forms. The fabrication of synthetic biocompatible scaffolds that can mimic the natural extracellular matrices is a useful activity for tissue engineering; hence, an effort has been made to develop 3D scaffolds by stacking the PLGA microfibrous meshes in alternate fashion with nanofibrous meshes of collagen using dual electrospinning technique. Since electrospinning technique is able to prove a significant control on the orientation and fiber diameter [55] in the scaffolds, hence electrospinning has been used in the fabrication of scaffolds by placing microfibrous PLGA meshes in alternate fashion with nanofibrous meshes of collagen. The bioactivity of layered scaffolds with different densities of nanofibrous collagen has been evaluated in comparison to pristine microfibrous PLGA scaffolds. The alternate patterning of microfibrous PLGA meshes with micro/nano mixed fibrous meshes of PLGA and collagen in the scaffolds has been designed to facilitate cell infiltration and to enhance the surface area for cells adhesion, proliferation, and differentiation [56].

## Methods

Poly(lactic-co-glycolic acid) with a weight ratio of lactic acid to glycolic acid of 85:15 (MW: 240 Da), 1,1,1,3,3,3-hexafluoroisopropanol, l-glutamic acid, 1-ethyl-3-(-3-dimethylaminopropyl dicarbodiimide hydrochloride) (EDC), n-hydroxysuccinamide (NHS), and 3-(4,5-dimethylthiazol-2-yl)-2,5-diphenyltetrazolium bromide (MTT) were purchased from Sigma-Aldrich Chemical Company, USA, and used without further purification. Collagen type 1 was purchased from Bioland Company, Korea. The 5(6)-tetramethyl-rhodamine isothiocyanate-conjugated phalloidin (TRITC) was purchased from Millipore, Billerica, MA, USA. The hydroxyapatite (C_10_(PO_4_)_6_(OH)_2_) as nanorods having suitable morphology, size, and clinical property was prepared in the laboratory using ammonium dihydrogen phosphate ((NH_4_)H_2_PO_4_) and calcium nitrate (Ca(NO_3_)_2_4H_2_O). The mouse pre-osteoblast cells (MC3T3-E1) were purchased from Korea cells bank, Seoul, South Korea, and stored in liquid nitrogen before carrying out cell seeding experiments. The α-minimum essential medium (α-MEM), 10 % fetal bovine serum (FBS), and penicillin G-streptomycin were purchased from Gibco, Tokyo, Japan. The cells were cultured in α-MEM containing 10 % FBS and 1 % antibiotic. The alkaline phosphatage (ALPage), alizarin red staining kits, and 4′,6-diamidino-2-phenylindole (DAPI) were purchased from Millipore, Billerica, MA, USA. Triton X-100 and 10 × 10^−3^ mmol phosphate-buffered saline (PBS) solution (pH 7.4) containing 87 × 10^−3^ mmol Na_2_HPO_4_, 14 × 10^−3^ mmol KH_2_PO_4_, 131 × 10^−3^ mmol NaCl, and 27 × 10^−3^ mmol KCl was purchased from Sigma-Aldrich Chemical Company, USA. Other chemicals and solvents used in the experimental work were of high purity reagents and purchased from Sigma-Aldrich Chemical Company, USA. The multilayered scaffolds with microfibrous meshes of PLGA and multilayered scaffolds with nanofibrous meshes of collagen were electrospun by using single electrospinning technique. The multilayered scaffolds with microfibrous meshes of PLGA in sequence with micro/nano mixed fibrous meshes of PLGA and collagen were fabricated using dual extrusion electrospinning technique.

### Synthesis of Hydroxyapatite Nanorods

The nHA of controlled size and morphology [57] were prepared by using a method of chemical precipitation as reported in previous communication [34]. Briefly, 400 mL solution of (NH_4_)H_2_PO_3_ and 300 mL solution of Ca(NO_3_)_2_4H_2_O were prepared separately by dissolving 19.75 g of (NH_4_)H_2_PO_3_ and 57.5 g of Ca(NO_3_)_2_4H_2_O in 400 and 300 mL of deionized water, respectively. Before dropwisely mixing the solution of (NH_4_)H_2_PO_3_ with Ca(NO_3_)_2_4H_2_O, the pH of Ca(NO_3_)_2_4H_2_O solution was adjusted to 10.4 by adding an adequate amount of NH_4_OH. After dropwise addition of the total amount of (NH_4_)H_2_PO_3_, the solution was stirred vigorously at room temperature for about 1 h for proper mixing of the reactants. On keeping the mixture, a gelatinous white precipitate was obtained, which ultimately seeded to nHA after ageing for 4 days. The prepared nHA were separated and washed gently with double-distilled water until pH 7. Before vacuum evaporation by drying process, the separated nHA were suspended in 1-butanol to avoid clustering of nHA on drying. After vacuum evaporation, nHA were dried at 80 °C in vacuum oven to remove the traces of solvent and finally annealed at 700 °C for 4 h in hot air oven. To confirm the formation of nHA, FT-IR (FT-IR Spectrophotometer Mattason, Galaxy 7020 A) and X-ray photoelectron spectra (ESCA, ESCA LAB VIG Microtech, Mt 500/1, Etc. EAST Grinstead, UK) were recorded.

### Synthesis of Glutamate-Functionalized Hydroxyapatite Nanorods

**Fig. 1:**
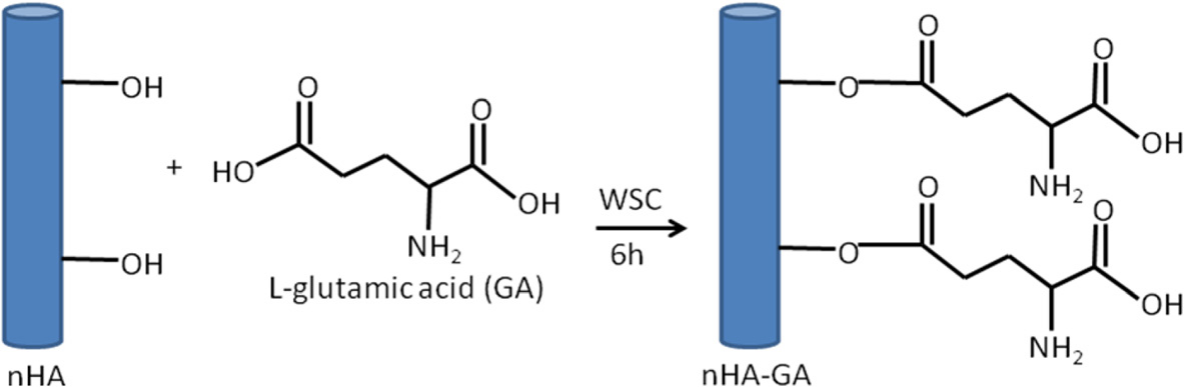
Surface modification of HA nanorods by l-glutamic acid

To enhance the dispersion of nHA in collagen fiber, the surface of nHA was modified with l-glutamic acid (GA) [58]. The excess amount of GA (500 mg, 4.27 mmol) was dissolved in 10 mL PBS solution (pH 7.4), and the carboxylic acid group of GA was activated by adding 10 mL solution of EDC (200 mg, 0.64 mmol) and NHS (200 mg, 1.74 mmol) under stirring conditions and the mixture was kept for 8 h at 5 °C. After activating GA, 10 mL PBS solution containing 500 mg nHA was added and allowed to react with activated GA for 6 h in ice-cooled vessel under constant stirring. Finally, reaction mixture was dialyzed using regenerated cellulose membranes till pH 7.0. The modified nHA after washing with PBS was lyophilized to obtain white powdery solids of GA-functionalized nHA (nHA-GA) (Fig. 1).

To confirm the anchoring of GA on nHA, FT-IR (FTIR-Spectrophotometer, Mattason, Galaxy 7020A) and X-ray photoelectron spectra (ESCA, ESCA LAB VIG Microtech, Mt 500/1, Etc. EAST Grinstead, UK) were recorded.

### Fabrication of Alternately Micro/Nano PLGA-Col-HA Mixed Fibrous Multilayered Scaffolds

**Fig. 2:**
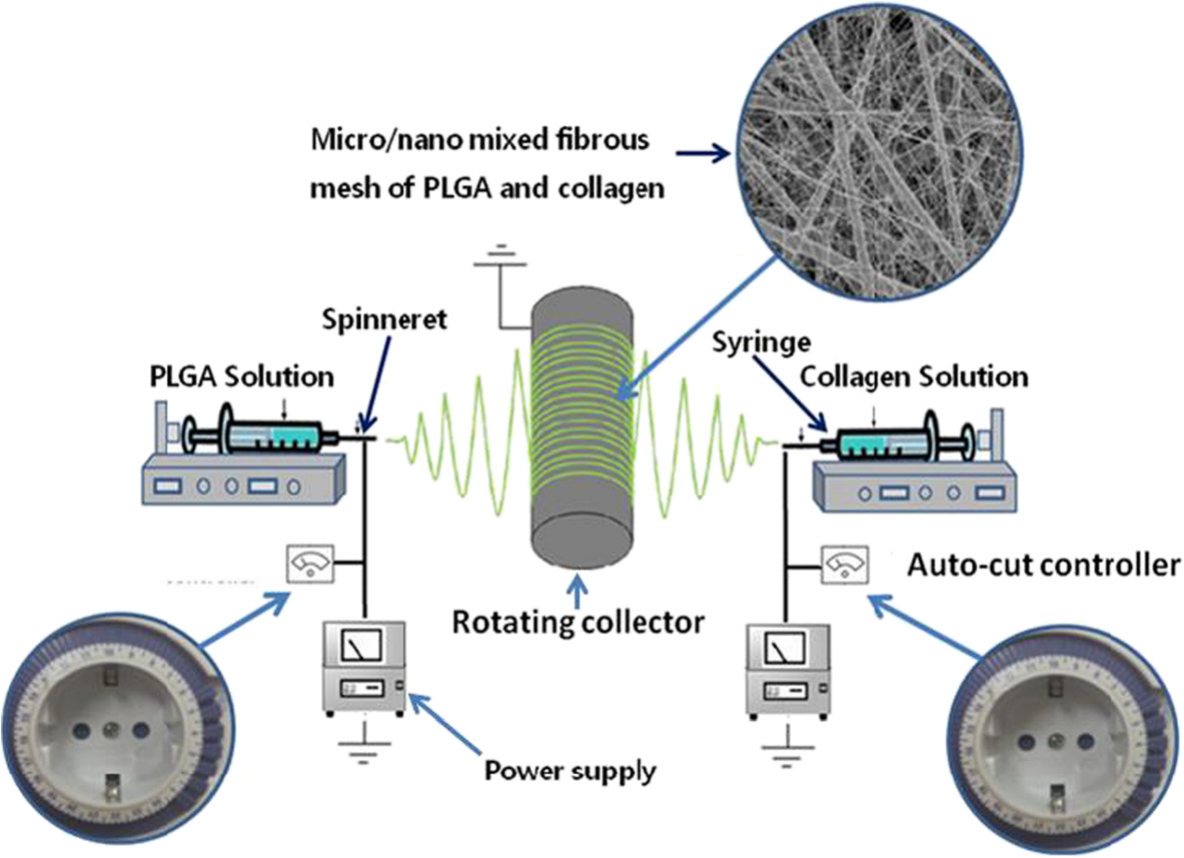
Schematic representation of dual electrospinning technique for fabrication of multilayered 3D scaffolds

To fabricate the multilayered scaffolds having sequential arrangement of microfibrous PLGA meshes and micro/nano mixed fibrous meshes of PLGA and collagen, the dual extrusion electrospinning technique was used (Fig. 2). The 35 wt% solution of PLGA in THF-DMF binary solvent (3:1) and the 5 wt% solution of collagen in HFIP with and without glutamate-functionalized nHA hydroxyapatite nanorods (nHA-GA) were prepared for individual electrospinning of PLGA and collagen meshes and for fabrication of multilayered 3D scaffolds of microfibrous PLGA meshes in alternate sequence with micro/nano mixed fibrous meshes of PLGA and Col-nHA-GA by dual extrusion electrospinning technique. The prepared solution of collagen (5 wt%) in HFIP was stirred magnetically for overnight. To prepare the solution of collagen-containing nHA-GA, 0.03 g of nHA-GA was sonicated in 5 mL solution of HFIP for about 10 h before dropwise mixing to 5 mL of 10 wt% solution of collagen in HFIP under stirring. The solution containing collagen and nHA-G was stirred further overnight for proper mixing and dispersion of nHA-GA without getting any kind of aggregates and sedimentation before electrospinning.

**Fig. 3:**
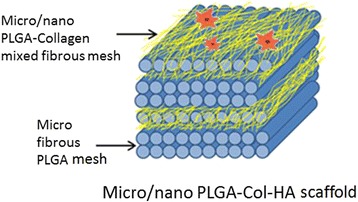
Schematic representation of the structure and composition of multilayered scaffolds having micro/nano mixed fibrous PLGA and Col-HA mesh arranged in alternate fashion with microfibrous PLGA mesh as fabricated with dual extrusion electrospinning technique

The PLGA solution was prepared by adding 3.5 g of PLGA in 10 mL binary mixture of THF and DMF (3:1) under vigorous stirring. The solution of PLGA was kept stirred overnight similar to nHA-GA dispersed solution of collagen. Subsequently, these solutions of PLGA and nHA-GA-containing collagen (Col-nHA-GA) were taken in two different 10 mL glass syringes fitted with 20-gauge needles having an inner diameter of 0.90 mm. The syringes containing the solution of PLGA and collagen were loaded on two separate syringe pumps, which were placed across the collecting spinning drum of electrospinning system (Fig. 2). The flow rates of PLGA and collagen solutions were controlled to 1 and 0.5 mL/h, respectively, by syringe pumps. After optimizing, the electrospinning conditions, the tip-to-drum distances for electrospinning of PLGA and collagen solutions were fixed at 10 and 15 cm, respectively. For the fabrication of individual and mixed fibrous meshes of multilayered scaffolds, the dual extrusion electrospinning technique was applied using intermittent auto-cuts for electrospinning of collagen nanofibers. The dual extrusion electrospinning system was placed in a humidifier, and humidity was maintained to 55 % at room temperature. To start the electrospinning, the syringe needles were connected to the positive terminal of a high-voltage power supply (Gamma High Voltage Ormond, Beach, Florida, USA), and the rpm of the collecting drum was fixed to a speed of 500 rpm for electrospinning of multilayered sequential scaffolds. The electrospinning was started when high electric current was generated on application of 20 kV. These solutions were electrospun simultaneously on the same targeting drum for a total time of 6 h. After electrospinning, the scaffolds were left overnight for drying. To obtain scaffolds with different structures and compositions, the flow of collagen solution was auto-cut intermittently from 6 to 14 times with a cutoff time of 5 min each. The simultaneous electrospinning parameters for PLGA and collagen solutions were optimized by individual electrospinning of PLGA and collagen solutions. The morphology and composition of mixed fibrous meshes in the multilayered scaffolds were controlled by varying electrospinning parameters of individual fibers (Fig. 3). The individual and mixed meshes of microfibrous PLGA and nanofibrous collagen in multilayered scaffolds were fabricated at optimized electrospinning parameters (Table 1). To make a visible difference between collagen and PLGA fibers in micro/nano mixed fibrous meshes of multilayered scaffolds, the collagen solution containing FITC (500 mg) was also prepared and used in the fabrication of alternately arranged micro/nano mixed fibrous meshes of PLGA and collagen in multilayered scaffolds. After dual extrusion electrospinning, the scaffolds were removed carefully from the electrospinning drum and dried overnight at 40 °C to remove the residual solvent.

**Table 1:** Optimized dual electrospinning parameters for individual and mixed fibers meshes

Materials	Amount/wt%	Solvent	Flow rate/mLh^−1^	Tip-to-drum distance/cm	Voltage/kV
Collagen	5.0	HFIP	0.5	20	20
PLGA	35	THF-DMF	1.0	15	20

Humidity 55 %

### Characterization of Alternately Micro/Nano PLGA-Col-HA Mixed Fibrous Multilayered Scaffolds

The individual and mixed fibrous scaffolds were fabricated by individual and simultaneous electrospinning of solutions of PLGA and collagen with nHA-GA. The fabricated scaffolds were characterized by recording FE-SEM micrographs (FE-SEM, 400 Hitachi, Tokyo, Japan) to evaluate the effect of solution properties of PLGA and collagen and electrospinning parameters on the morphology, fiber diameter, and architectures of mixed fibrous scaffolds. To record the FE-SEM micrographs, 2 × 2 mm-sized samples of scaffolds were cut and placed on metal stubs using double-adhesive tapes before sputter-coating with gold. The FE-SEM images were recorded for individual and mixed fibers scaffolds fabricated in the presence of nHA-GA nanorods. To visualize the size and distribution of PLGA and collagen fibers in mixed fibrous meshes of multilayered scaffolds, the confocal laser scanning micrographs were also recorded (Zeis LSM410, Zeiss, Oberkoshen, Germany). To visualize the size and distribution of nHA-GA in collagen fibers, the TEM micrographs of collagen fibers were recorded (TEM, H-7600, Hitachi, Japan) using carbon-coated 200-mesh copper grids. The presence of nHA-GA and collagen in multilayered scaffolds is confirmed by recording FT-IR spectra (FTIR Mattason, Galaxy 7020A). To record the FT-IR spectra, the samples of nHA and nHA-GA were ground and mixed with KBr to prepare pellets using hydraulic press, whereas the FT-IR spectra of the scaffolds were recorded using ATR-FTIR. The X-ray photoelectron spectra (XPS) of individual and mixed fibers scaffolds with and without nHA-GA is also recorded (ESCA, ESCA LAB VIG Microtech, Mt 500/1, Etc. EAST Grinstead, UK) for surface mapping of elements and to confirm the type of polymers used in fabrication of individual (collagen/PLGA) and mixed fibrous scaffolds. The XPS spectra were recorded using Mg Kα radiation at 1 and 253.6 ev and 150 W power supply at anode.

### In Vitro Cell Culture

To determine the effect of composition and structures of multilayered scaffolds on the bone-forming activity of osteoblast, the circular-shaped samples of scaffolds were cut and fitted in a 24-well culture dish for cell seeding after sterilization with UV irradiation for 2 h. Five hundred microliters of non-osteogenic α-minimum essential medium (α-MEM: Gibco, Tokyo, Japan) supplemented with 10 % fetal bovine serum and 1 % penicillin/streptomycin was added in each well, and then MC3T3-E1 cells were seeded at a cells density of 3 × 10^4^ cells/cm^2^ per sample of the scaffolds. The scaffolds seeded with cells were incubated at 37 °C in the presence of 5 % CO_2_ for 3 days to evaluate the adhesion of the cells to the scaffolds having different structures and compositions. The medium was changed every second day if cells were seeded for more than 1 day. After incubation, the supernatant medium was removed to Eppendorf tubes carefully and scaffolds were washed twice with PBS solution before fixing with an aqueous solution of 2.5 % glutaraldehyde for 20 min. Finally, scaffolds were dehydrated with critical point drier (EMS 850 Critical Point Dryer, Hatfield, PA, USA) and stored after drying to record their FE-SEM (400-Hitachi, Tokyo, Japan) micrographs.

### MTT Assay

To analyze the bioactivity of scaffolds for proliferation of MC3T3-E1 cells, MTT assay has been carried out by estimating the amount of purple formazan produced by mitochondrial reduction of thiazolyl blue tetrazolium bromide at different times of cell seeding on scaffolds. For MTT assaying, the sterilized samples of scaffolds were fitted in a 24-well dish and after adding 500 μL of non-osteogenic α-minimum essential medium, MC3T3-E1 cells were seeded at a density of 3 × 10^4^ cells/cm^2^ per scaffold. After incubation for 3 days, the supernatant medium was removed carefully and scaffolds were washed twice with PBS solution. The cell-seeded scaffolds were incubated with 500 μL of 500 μg/mL solution of MTT for 4 h at 37 °C and the supernatant solution was discarded. The formazan purple crystals produced were extracted by adding 250 μL of dimethyl sulfoxide (DMSO, Sigma-Aldrich Chemical Company, USA) to each well for 10 min. The wells seeded with MC3T3-E1 cells in the absence of scaffolds were treated as positive control, and the empty wells without cells were used as negative controls. The absorbance of extract was recorded at 570 nm with reference to 690 nm for the medium using Symergy HT multidetection microplate reader (Symergy HT, BioTek, USA). The amount of formazan so produced is determined by using microplate reader data. The data obtained from negative controls were subtracted from measured values. The number of viable cells was correlated to the optical density, and cell viability was then evaluated by normalizing the values to those from the positive control wells.

### Alkaline Phosphatase Activity

The differentiation of MC3T3-E1 cells on the scaffolds was estimated with the expression of alkaline phosphatase (ALP) activity; the MC3T3-E1 cells were seeded in a 24-well dish at a cell density of 3 × 10^4^ cells/cm^2^ on sterilized scaffolds in α-minimum essential medium for 15 days. ALP staining was performed by a standard procedure according to the manufacturer’s instructions (Sigma-Aldrich Chemical Company, USA). After culturing, MC3T3-E1 cells were washed with deionized water and fixed with a citrate-acetone-formaldehyde fixative solution (citrate solution 25 mL, acetone 65 mL, and 8 mL 37 % formaldehyde solution) for 30 s. Subsequently, the cell-fixed discs were rinsed three times with deionized water for 45 s and stained with alkaline-dye mixture (Fast Blue RR salt solution 48 mL, naphthol AS-MX phosphate alkaline solution 2 mL) at room temperature for 30 min and the immersed slides were protected from direct light. After removing the dye solution, the dyed samples were rinsed three times with deionized water for 2 min to completely remove the redundant stains and then dried. After rinsing, the discs were placed in Mayer’s hematoxylin solution for 10 min. The cells stained positively for ALP were observed with an optical microscope (Nikon E 4500, Japan).

### Alizarin Red Staining

To evaluate the mineralization and cell differentiation capacity of the prepared multilayered mixed fibrous scaffolds of PLGA and collagen, the MC3T3-E1 cells were seeded in a 24-well dish at a cell density of 3 × 10^4^ cells/cm^2^ on sterilized scaffolds in α-minimum essential medium for 15 days by changing the medium every two alternate days. At the end of 15 days, the medium was aspirated gently without disturbing the grown cells on the scaffolds. The cell-seeded scaffolds were washed twice with PBS solution before fixing with an aqueous solution of 10 % formaldehyde for 15 min at room temperature. After fixing cells on scaffolds, the fixative solution was removed carefully from the wells and cell-seeded scaffolds were washed with distilled water three times with a time interval of 10 min each. On complete removing the water from each well, 1 mL of 10 wt% solution of Alizarin Red S (Sigma-Aldrich Chemical Company, USA) was added to each well and scaffolds seeded with cells were stained with alizarin red for 30 min at pH 4.2. On the completion of staining, the excess amount of Alizarin Red was removed from the wells and scaffolds were washed with distilled water until colorless washing was obtained. Finally, the stained scaffolds were examined under microscope (Nikon E 4500, Japan) and digital images were captured.

### von Kossa Assay

To estimate the calcium deposition of MC3T3-E1 cells on scaffolds of different structures and composition, the von Kossa staining was carried out by culturing pre-osteoblast MC3T3-E1 cells on scaffolds for 15 days in 24-well dish following the steps as used in Alizarin Red staining. The cell-seeded scaffolds after washing three times with PBS for 5 min were fixed with 10 % formaldehyde for 30 min. The fixed scaffolds were again washed three times with distilled water for 10 min. The fixed scaffolds were then treated with 5 % solution of AgNO_3_ and exposed to UV irradiation for 5 min. The UV irradiated scaffolds were washed two times with PBS to remove unused AgNO_3_ and kept in 5 % solution of Na_2_S_2_O_3_ for 5 min. Finally, the scaffolds were washed twice gently with distilled water and digital images of stained cells were captured by a microscope (Nikon E 4500, Japan) fitted with camera.

### Actin Cytoskeleton Assay

After evaluating the mineralization activity of scaffolds in osteogenic differentiation of MC3T3- E1 cells, the actin cytoskeleton organization of scaffold seeded with cells is also assayed to evaluate the effect of scaffolds on osteogenesis. The MC3T3-E1 cells were incubated for 3 days on scaffolds in a 4-well dish following the steps as used in von Kossa and Alizarin Red assays. After 7 days, the cell-seeded scaffolds were washed with PBS and permeabilized with 0.5 % solution of formaldehyde and kept in a PBS solution (pH 7.4) containing 0.2 % Triton X-100 for 5 min at room temperature. After permeabilization, the scaffolds were washed with PBS and fixed using 4 % formaldehyde in PBS for 20 min. After three times washing with PBS, the scaffolds were incubated in a PBS containing 1.0 % bovine serum albumin for 30 min to block non-specific binding sites of antibody. After three times rinsing with PBS, the scaffolds were stained using fluorescent 5(6)-tetramethyl-rhodamine isothiocyanate-conjugated phalloidin in PBS for 1 h at room temperature to visualize the actin cytoskeletal filaments (F-actin) of the cells. After washing with PBS three times (10 min each), the scaffolds were stained with fluorescent DAPI by incubating for 5 min in PBS to visualize the nuclei of the cells.

### Statistical Analysis

All data are presented as means ± standard deviations. Experiments were carried out in triplicates, and statistical analyses were performed using Student’s two tailed test in conjunction with Scheffe’s test for multiple comparison statistics considering *p* < 0.05, *P* < 0.01, and *P* < 0.001 as statistically significant, very significant, and extremely significant values, respectively, whereas *P* > 0.05 is treated as statistically insignificant value.

## Results and Discussion

The osteogenic properties of various scaffolds have largely shown dependence on the types of polymers [58, 59] used in their fabrication. The properties of the scaffolds have also shown significant variations with the types of ceramics embedded in the matrices. The application of ceramics as nanorods is found to be more promising as we have reported in our previous studies [33, 34] and by other workers [60]. The fabrication of multilayered mixed fibrous scaffolds using dual extrusion electrospinning technique has provided an opportunity to utilize the properties of both collagen and PLGA together in designing scaffolds with different structures and bioactivities. The technique has provided ample opportunity to utilize nanofibrous collagen in combination with microfibrous PLGA and embedded bioactive nanorods of hydroxyapatite. To fabricate mixed fibrous scaffolds having homogeneously distributed nHA in collagen, the surface fictionalization of nHA is proved to be potentially useful as indicated by the enhanced bioactivity of the fabricated 3D scaffolds.

### Functionalization of Hydroxyapatite Nanorods with l-Glutamic Acid

**Fig. 4:**
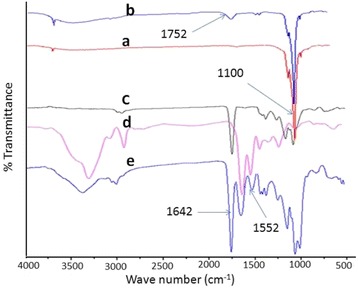
FT-IR spectra of hydroxyapatite nanorods without **(a)** and with glutamic acid **(b)**, FT-IR spectra of microfibrous meshes of PLGA **(c)**, nanofibrous meshes of collagen **(d)**, and hydroxyapatite nanorods containing collagen fibers in micro/nano mixed fibrous meshes of PLGA and collagen **(e)**

Amongst the various ceramics, the hydroxyapatite is found to be more osteogenic and its activity is found to vary with the shape and size of hydroxyapatite particles [33]. In previous studies, we have been able to reveal that hydroxyapatite as nanorods was suitably dispersed in nanofibrous composite of PLGA and shown enhanced osteogenic response for MC3T3-E1 cells in comparison to spherically shaped nanoparticles of hydroxyapatite [33, 34]. Therefore, hydroxyapatite has been used as nanorods in the present studies. The hydroxyapatite nanorods were synthesized using solutions of dihydrogen ammonium phosphate ((NH_4_)H_2_PO_3_) and calcium nitrate (Ca(NO_3_)_2_4H_2_O) at pH 10.4. The formation of hydroxyapatite was confirmed by FT-IR spectra and TEM micrographs. To enhance the dispersity and mixing of nHA in collagen, the surface of the nHA was modified by reacting GA in the presence of activating agent (Fig. 1). To confirm the immobilization of GA on nHA, the FT-IR spectra of pristine nHA and nHA-GA were recorded. The FT-IR spectrum of nHA has shown a characteristic peak of phosphate at 1100 cm^−1^ (Fig. 4a).

The FT-IR spectra of nHA-GA has shown characteristic absorption bands at 1652 and 3500 cm^−1^ corresponding to carboxylic and hydroxyl groups of GA on nHA, respectively (Fig. 4b). The presence of collagen fibers and nHA in multilayered mixed fibrous scaffolds was also confirmed by FT-IR spectra of microfibrous meshes of PLGA (Fig. 4c), nanofibrous collagen (Fig. 4d), and micro/nano mixed fibrous meshes of PLGA and collagen-containing hydroxyapatite nanorods (Fig. 4e). The presence of absorption band corresponding to the phosphate group (–PO_3_) of hydroxyapatite at around 1100 cm^−1^ and absorption band at 1642 cm^−1^ for stretching vibration of the carbonyl group of amide I (–CO–NH–) and at 1552 cm^−1^ for coupling of –NH bending and –C–N stretching vibration of amide II confirmed the presence of collagen [61] and nHA in mixed fibrous meshes of PLGA and collagen (Fig. 4e). The nanofibrous meshes of collagen have shown absorption bands (Fig. 4d) as found in FT-IR spectra of mixed fibrous meshes (Fig. 4e).

The absorption band around 1760 cm^−1^ for stretching frequencies >C=O groups of pure PLGA (Fig. 4c) also appeared in FT-IR spectrum of mixed fibrous meshes (Fig. 4e), which confirmed the presence of PLGA in mixed fibrous meshes (Fig. 4e).

**Fig. 5:**
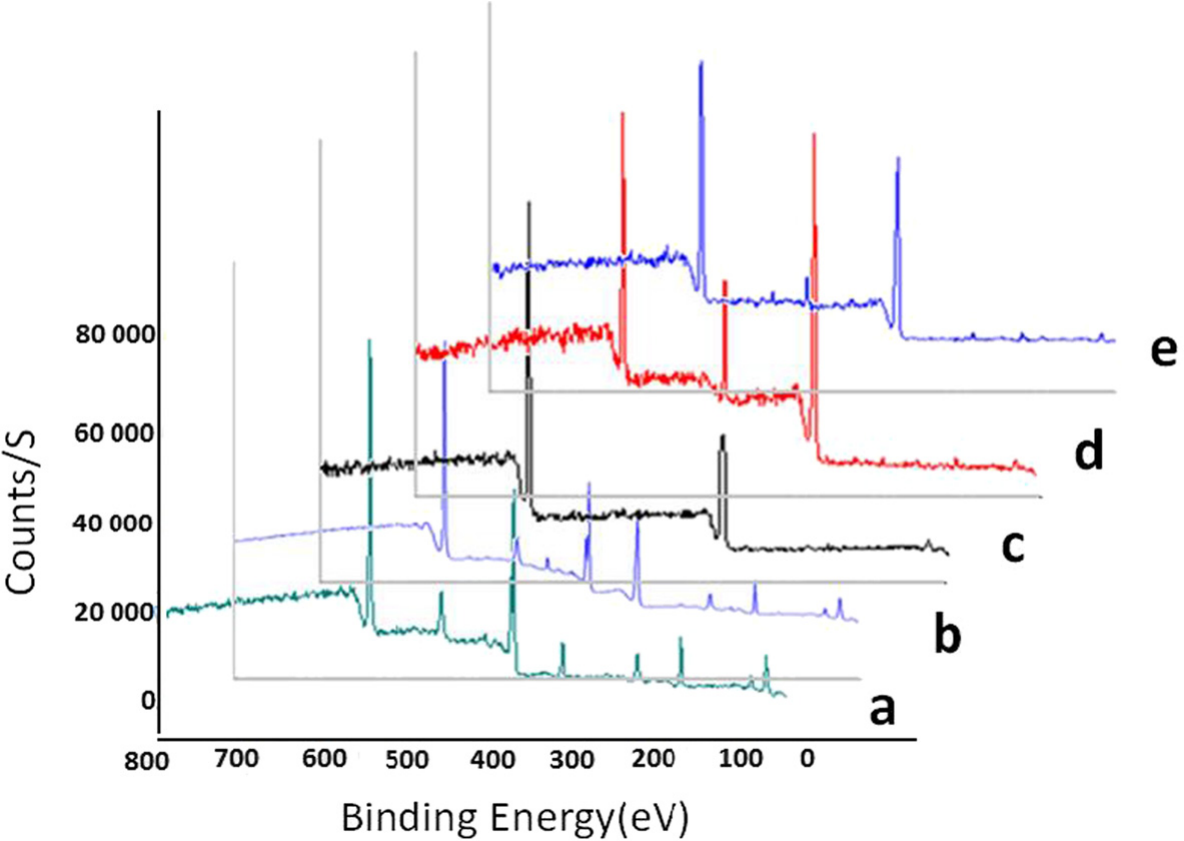
X-ray photoelectron spectra of hydroxyapatite nanorods without **(a)** and with glutamic acid **(b)**, PLGA **(c)**, collagen **(d)**, and mixed fibrous multilayered scaffolds of PLGA and Col-HA **(e)**

In addition to FT-IR characterization, the nHA and nHA-GA were also characterized by X-ray photoelectron spectra (Fig. 5). The X-ray photoelectron spectra of nHA have confirmed the presence of constituent elements of hydroxyapatite. The decrease in percentage of calcium from 17.86 to 12.11 % and phosphorous from 12.78 to 11.14 % in X-ray photoelectron spectra of nHA-GA has confirmed the anchoring of GA on nHA (Table 2). The presence of new peak of nitrogen in X-ray photoelectron spectra of nHA-GA has further confirmed the anchoring of GA on nHA (Fig. 5a, b). The complete agreement of XPS (Fig. 5, Table 2) and FT-IR (Fig. 4a, b) data have confirmed the anchoring of GA on nHA. The X-ray photoelectron spectrum recorded for the survey of elements in nHA-GA has shown 2.16 % for nitrogen, which has confirmed the presence of GA on nHA (Fig. 5b, Table 2).

**Table 2:** X-ray photoelectron data for the survey of the elements in the scaffolds

Substrates	Amount of element (%)				
	C	O	Ca	N	P
nHA	7.52	66.54	17.86		12.78
nHA-GA	8.10	67.49	12.11	2.16	11.14
PLGA	67.29	32.71	–	–	–
Collagen	71.25	16.17	–	12.59	–
PLGA-Col-HA	64.51	24.73	1.20	8.48	1.08

The X-ray photoelectron spectra of mixed fiber meshes with nHA has shown 8.48 % of nitrogen corresponding to N1s peak (Fig. 5e, Table 2). This result has supported the presence of collagen fibers in mixed fiber matrices. The decreasing percent of nitrogen from 12.59 to 8.48 % has supported the dilution of collagen densities in mixed fibers meshes by PLGA and nHA. The area for C1s and O1s peaks corresponding to 64.51 and 24.73 % have also supported the presence of collagen and PLGA together in mixed fiber meshes (Fig. 5e, Table 2). The appearance of Ca ^2^P_3/2_ and Ca ^2^p_1/2_ peaks in X-ray photoelectron spectra of mixed fiber meshes corresponding to 1.20 % of calcium has confirmed the presence of nHA (Fig. 5e). The presence of P ^2^p with an area corresponding to 1.08 % phosphorous in X-ray photoelectron spectrum of mixed fibers meshes (Fig. 5e) has also supported the presence of hydroxyapatite nanorods in the mixed fiber meshes of the multilayered scaffolds.

### Fabrication and Characterization of Alternately Micro/Nano Multilayered Scaffolds

**Fig. 6:**
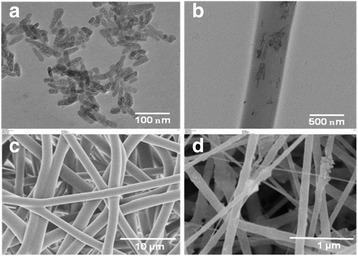
TEM images of hydroxyapatite nanorods **(a)** and their dispersion in collagen fiber **(b)**. SEM micrographs of PLGA **(c)** and Col-HA **(d)**

The electrospinning technique has been used to fabricate the multilayered scaffolds consisting of microfibrous meshes of PLGA and micro/nano mixed fibrous meshes of PLGA and collagen after optimizing the electrospinning conditions and solution parameters for electrospinning of individual meshes of collagen and PLGA. To enhance the cells adhesion and proliferation on multilayered scaffolds of microfibrous meshes of PLGA, and micro/nano mixed fibrous meshes of PLGA and collagen, the hydroxyapatite as nanorods (nHA) was incorporated into collagen nanofibers. To confirm the shape, size, and distribution of nHA in collagen nanofibers, the TEM images of the scaffolds were recorded as shown in Fig. 6. The TEM images have clearly indicated that the hydroxyapatite particles mostly were in the shape of nanorods with a size variation from 60 to 80 nm in length and 10–20 nm in their diameters (Fig. 6a), and they were distributed randomly in the bulk of collagen nanofibers (Fig. 6b). The shape, size, and distribution of nHA in collagen nanofibers have contributed significantly in controlling the bioactivity of sequentially arranged microfibrous meshes of PLGA with micro/nano mixed fibrous meshes of PLGA and collagen in fabricated multilayered scaffolds as found in the previous study [33]. The 5 wt% solution of collagen in HFIP at a flow rate of 0.5 mL/h without nHA and with 5 wt% nHA has produced nanofibrous meshes. The FE-SEM micrographs (Fig. 6c, d) of nanofibrous scaffolds of collagen have indicated that the diameter of collagen fibers has varied from 200 to 400 nm. The collagen nanofibrous meshes fabricated with 5 wt% solution of collagen and 5 wt% of nHA have shown a random distribution of nHA at the surface and bulk of collagen nanofibers as clear from FE-SEM (Fig. 6d) and TEM micrographs (Fig. 6b).

**Fig. 7:**
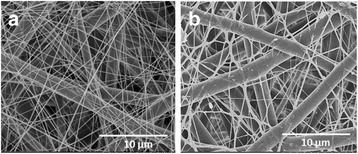
FE-SEM micrographs for micro/nano mixed fibrous scaffolds of PLGA and Col **(a)** and PLGA and Col-HA **(b)**

The multilayered scaffolds having microfibrous meshes of PLGA (Fig. 6c) and mixed meshes of microfibrous PLGA and nanofibrous collagen were fabricated using dual electrospinning technique (Fig. 7a, b). The FE-SEM micrographs have clearly indicated that 35 wt% solution of PLGA has produced microfibrous meshes, whereas 5 wt% solution of collagen has produced nanofibrous meshes as clear from the mixed fibrous meshes of PLGA and collagen (Fig. 7a, b). Therefore, multilayered scaffolds having microfibrous meshes of PLGA and micro/nano mixed fibrous meshes of PLGA and collagen arranged in sequence were fabricated using 35 wt% solution PLGA and 5 wt% solution of collagen. The multilayered scaffolds fabricated using 5 wt% solution of collagen with 5 wt% nHA (Fig. 7b) were found to be more bioactive. The FE-SEM micrographs of multilayered scaffolds of mixed meshes have shown a distribution of nHA at the surface of collagen nanofibers (Fig. 7b), which might be considered responsible for enhanced bioactivity of the scaffolds [62] in comparison to individual microfibrous meshes of PLGA (Fig. 6c) and nanofibrous meshes of collagen (Fig. 6d). To fabricate micro/nano mixed fibrous meshes sandwiched with microfibrous meshes of PLGA, the flow of collagen solution was programmed to auto-cuts in dual extrusion electrospinning technique for a fixed time interval of 5 min. The number of auto-cuts in a total time of 6 h for dual extrusion electrospinning has controlled the overall composition of multilayered scaffolds (Figs. 7 and 2).

**Fig. 8:**
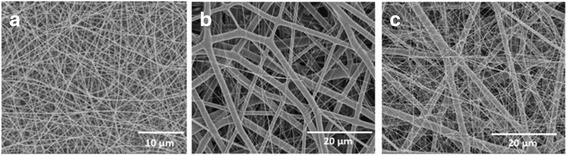
FE-SEM micrographs of nanofibrous meshes of collagen **(a)** and PLGA and collagen mixed fibrous meshes with low **(b)** and high **(c)** densities of collagen fibers

To vary the densities of collagen nanofibers in multilayered mixed fibrous scaffolds, the scaffolds were fabricated by varying the number of auto-cuts for electrospinning of collagen fibers in dual extrusion electrospinning from 6 to 14 times within a total spinning time of 6 h. The multilayered scaffolds, which were obtained by auto-cutting the flow of collagen solution to 14 times, have produced multilayered scaffolds with low densities of collagen (Fig. 8b), whereas scaffolds fabricated by auto-cutting the solution of collagen for 6 times have produced the multilayered scaffolds with high densities of collagen (Fig. 8c). The microfibrous PLGA meshes in the scaffolds have provided a mechanical strength and supported the overall proliferation of seeded cells (MC3ET3-E1).

**Fig. 9:**
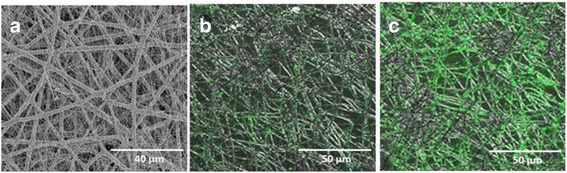
FE-SEM micrograph of micro/nano mixed fibrous meshes of PLGA and high-density collagen **(a)** and CLSM micrographs of micro/nano mixed fibrous meshes of PLGA and low- **(b)** and high-density collagen fibers **(c)**

To visualize the density of collagen nanofibers in mixed meshes of microfibrous PLGA and nanofibrous collagen in multilayered scaffolds, the dual extrusion electrospinning was carried out using the solution of FITC-conjugated collagen. The confocal laser scanning microscope (Zeis LSM410, Zeiss, Oberkoshen Germany) has been used for imaging the density of FITC-conjugated collagen nanofibers in the mixed fibrous meshes of PLGA and collagen (Fig. 9b, c).

The confocal laser scanning micrographs (CLSM) of mixed fibrous meshes were also recorded, which were fabricated without using FITC-non-conjugated collagen (Fig. 9a). The intensity of green fluorescence (*λ*_ex_ = 495 nm) is found to be low for the scaffolds fabricated using 14 auto-cuts for the flow of collagen solution (Fig. 9b), whereas scaffolds obtained with 6 auto-cuts were highly fluorescent (Fig. 9c).

This has clearly indicated that the densities of collagen fibers have varied significantly on varying the number of auto-cuts for the flow of collagen solution in dual extrusion electrospinning technique (Fig. 9). Thus, dual extrusion electrospinning has provided ample opportunities in the fabrication of 3D scaffolds with desired architectures structures and compositions to control their bioactivity [63].

### Bioactivity of Microfibrous Meshes of PLGA and Alternately Electrospun Micro/Nano Mixed Fibrous Meshes of PLGA and Collagen

The ceramic such as hydroxyapatite is a well-known material to increase the osteogenic properties and surface wetting of nanofibrous scaffolds. However, the ultimate effect of the addition of hydroxyapatite in the scaffolds on cell adhesion, proliferation, and bone tissue formation is found to be dependent on the shape and size of the hydroxyapatite nanoparticles [33, 34]. The present investigations have also indicated that the mixed fiber matrices of biocompatible materials such as PLGA and collagen may also serve as potential constructs for bone tissue engineering due to the synergistic effect of these materials on bioactivity of the scaffolds when mixed in optimized proportions and their fibers are arranged suitable in the scaffolds. The dual extrusion electrospinning is found to be a potential technique in controlling the hierarchical structures and topology of the scaffolds to influence their bioactivity [63] in comparison to other techniques of formation of scaffolds for tissue engineering.

### Cell Proliferation

**Fig. 10:**
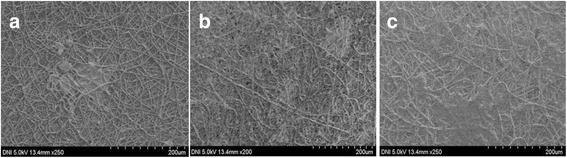
FE-SEM micrographs of osteoblasts cultured for 3 days on pristine PLGA **(a)** and mixed fibrous meshes of PLGA and Col **(b)** and PLGA and Col-HA **(c)**

The FE-SEM micrographs of MC3T3-E1 cell-seeded scaffolds have been used to evaluate the effect of hierarchical structure and composition of scaffolds on cell proliferations after incubation of 3 days in α-MEM. The proliferation of MC3T3-E1 cells on mixed fiber multilayered scaffolds is found to be significantly high (Fig. 10b, c), in comparison to pristine scaffolds of PLGA (Fig. 10a), which has clearly indicated that multilayered and mixed fibrous 3D scaffolds were able to provide suitable environments for proliferation to MC3T3-E1 cells in comparison to microfibrous scaffolds fabricated with pristine PLGA.

The nanofibrous collagen meshes intermingled with microfibrous meshes of PLGA were found to be more bioactive for proliferation to MC3T3-E1 cells (Fig. 10b, c) due to the presence of significantly high amount of nHA at the surface of nanofibrous meshes of the collagen in the scaffolds in comparison to the pristine microfibrous meshes of the PLGA (Fig. 10a). The seeded MC3T3-E1 cells were able to generate larger number of microvillar at collagen nanofibrous meshes for their proliferation (Fig. 10b, c) than at seeded on microfibrous meshes of PLGA [64]; hence, no significant proliferation of MC3T3-E1 cells was on microfibrous meshes of PLGA (Fig. 10a). This has clearly indicated that mixed fibrous meshes with nanofibrous collagen were having more cellular compatibility than pristine PLGA microfibrous matrices. The nanofibrous collagen in the mixed fibrous scaffolds has provided sufficient surface area and also contributed significantly toward interconnections [65] with adjacent layers of microfibrous meshes of PLGA in fabricated multilayered scaffolds. This has clearly confirmed that nanofibrous collagen has played a significant role in controlling the bioactivity of mixed fibrous multilayered scaffolds of PLGA and collagen. The electrostatic interactions between positively charged collagen and negatively charged MC3T3-E1 cells walls have also contributed significantly toward cell adhesion to nanofibrous collagen in mixed fibrous meshes of the multilayered scaffolds. The surface roughness of collagen nanofibers [66] due to the added nHA has also helped in the adhesion of the cells and their proliferation (Fig. 10b, c). The asymmetrical charge distribution on crystalline planes of nHA might have also played a significant role in MC3TE-E1 cell adhesion through electrostatic interactions between negatively charged surfaces of the cells with planner positively charge in the nHA [67, 68].

### MTT Assay

**Fig. 11:**
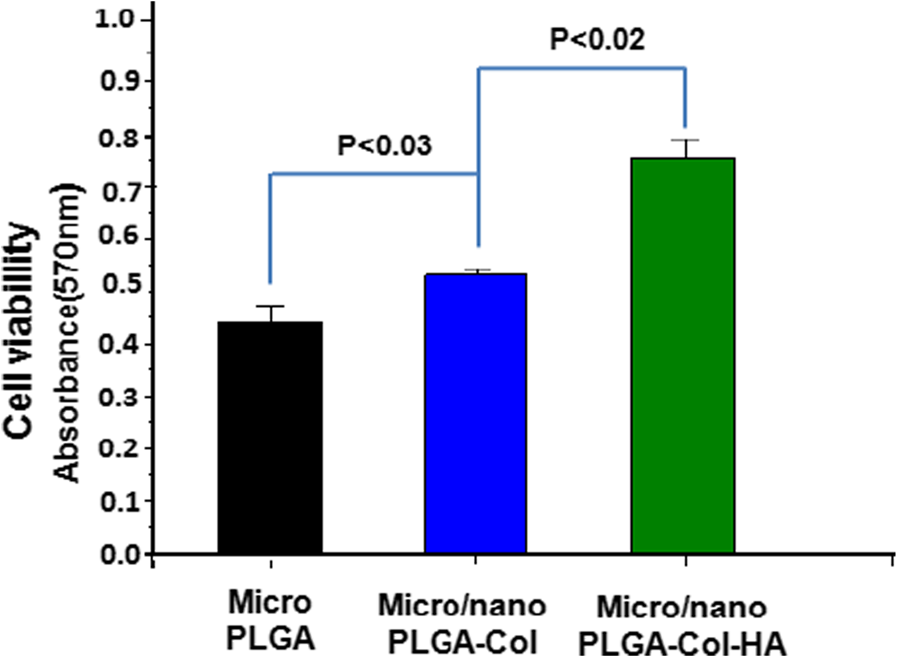
MTT assay of MC3T3-E1 cell cultured for 3 days on pristine PLGA and mixed fibrous meshes of PLGA and Col and PLGA and Col-HA

The cells’ viability and their rate of proliferation on bioactive scaffolds are assessed more accurately by applying MTT assay [69]. The MTT assaying for the viability of MC3T3-E1 cells on individual and mixed fibrous meshes of multilayered scaffolds has been carried out after incubating MC3T3-E1 cells (3 × 10^4^/cm^2^) for 3 days in α-MEM at 37 °C. The MC3T3-E1 cells’ viability without the scaffolds in the wells was taken as positive control, whereas viability without MC3T3-E1 cells was taken as negative control. The quantitative evaluation of cell viability was carried out by spectroscopic estimation of formazan (3-[4,5-dimethylthiazol-2-yl]-diphenyltetrazolium bromide) (*λ*_mx_ = 570 nm), which was produced on seeding of MC3T3-E1 cells on mixed fibrous meshes and microfibrous meshes of PLGA. The amount of formazan so produced is directly related to cell viability [70]. The MTT assay has indicated that cell viability has shown an increasing trend on increasing the time of cell seeding from 1 to 3 days. The cell viability has also increased with the increase in the density of nanofibrous collagen in the mixed fibrous meshes of multilayered scaffolds. In comparison to nanofibrous mesh of collagen and microfibrous meshes of PLGA, the viability of MC3T3-E1 cells on nano/micro mixed fibrous meshes of collagen and PLGA is found to be higher. The addition of nHA in the mixed fibrous meshes of collagen and PLGA has also produced positive effect on cell viability (Fig. 11).

The number of metabolically viable cells on microfibrous meshes of pristine PLGA, mixed meshes of microfibrous PLGA, and nanofibrous collagen without nHA and with nHA have shown an increasing trend (*P* < 0.03, *P* < 0.02) as shown in Fig. 11. The MC3T3-E1 cells proliferated more efficiently on mixed fiber meshes of multilayered scaffolds and on meshes having nHA-containing collagen. These results have confirmed the non-cytotoxicity of individual and mixed fibrous meshes of multilayered scaffolds fabricated by dual extrusion electrospinning of microfibrous meshes of PLGA and micro/nano mixed fibrous meshes of PLGA and collagen in the scaffolds.

### ALP Activity

**Fig. 12:**
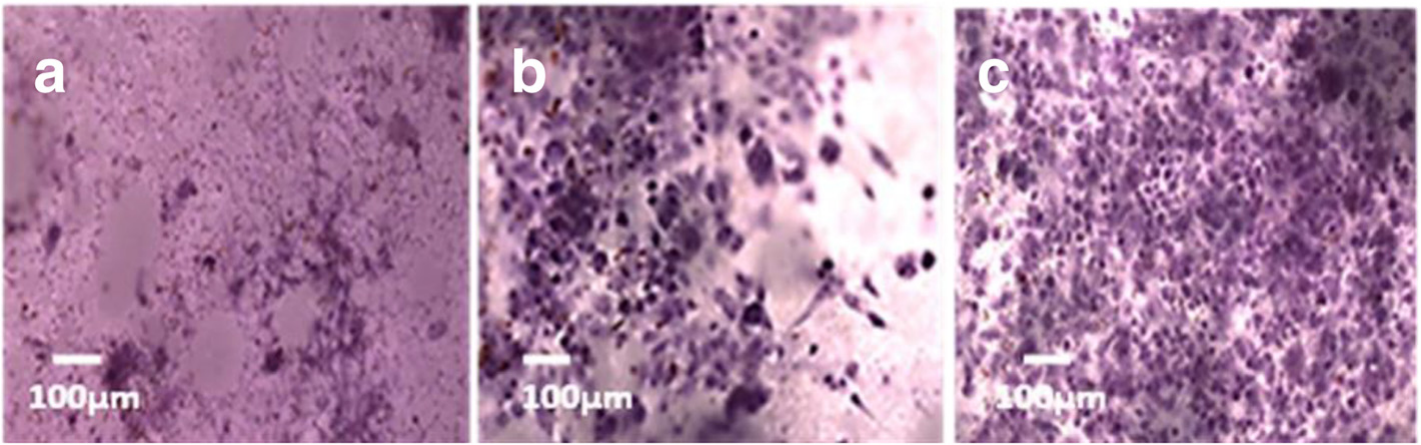
ALP activity of MC3T3-E1 cells on microfibrous PLGA scaffolds **(a)**, micro/nano mixed fibrous multilayered scaffolds of PLGA-Col **(b)**, and PLGA-Col-HA **(c)**

ALP activity of the scaffolds was determined as an indicator of osteoblastic differentiation of MC3T3-E1 cultured on microfibrous PLGA, mixed fibrous multilayered scaffolds of PLGA and Col, and PLGA and Col-HA [71]. The ALP expression is associated with formation of osteoprogenitors that proliferate and differentiate into identifiable osteoblasts, bone lining cells, and finally, to a new bone formation [72]. The polymerase chain reaction (PCR) is usually found useful in the early detection of gene-related markers expressed by osteoprogenitors. In the proliferation and differentiation states of the cells, the osteoblast-associated genes such as collagen type I (Coll-I), ALP, osteopontin (OSP), osteocalcin (OCN), and bone sialoprotein (BSP) are increased in a well-established temporal sequence with the development of osteoblast or bone formation. Therefore, as the cell differentiation increases, the increase in alkaline phosphatase activity takes place. Since the molecular finger printing of primitive osteoprogenitors needs PCR-related facilities, hence assaying of cell differentiation by alkaline phosphatase activity is reported in the present investigations. The degree of ALP activity expressed by MC3T3-E1 cells on mixed fibrous multilayered scaffolds of PLGA and Col-HA was found to be significantly high (Fig. 12c) in comparison to that of the microfibrous mesh of PLGA (Fig. 12a) and mixed fibrous multilayered scaffolds of PLGA and Col (Fig. 12b). It is clear that the addition of nHA enhanced the osteogenic properties of the mixed fibrous multilayered scaffolds (Fig. 12c) than without nHA.

This study has clearly indicated that mixed fibrous meshes of multilayered scaffolds were able to support the expression of collagenous gene, such as alkaline phosphatase, which is important for osteogenesis.

### Alizarin Red Staining

**Fig. 13:**
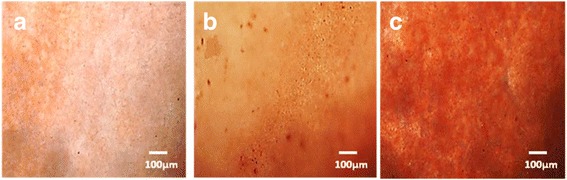
Alizarin Red staining MC3T3-E1 cell cultured for 15 days on microfibrous PLGA scaffolds **(a)**, micro/nano mixed fibrous multilayered scaffolds of PLGA and Col **(b)**, and PLGA and Col-HA **(c)**

The Alizarin Red staining has been used to indicate the process of transformation of undifferentiated MC3T3-E1 cells to osteoblasts and mineralization of scaffolds leading to the formation of bone [72]. The process of osteogenesis depends on the structure and properties of the scaffolds. The in vitro Alizarin Red staining was carried out to visualize the process of osteogenesis by recording the red color on the production of calcium. The osteoinductive and osteoconductive properties of the multilayered scaffolds were evaluated by Alizarin Red staining of MC3T3-E1 cell-seeded scaffolds fabricated with sequential stacking of microfibrous meshes of PLGA and micro/nano mixed fibrous meshes of PLGA and collagen (Fig. 13).

The Alizarin Red staining of the cells cultured for 15 days was carried out to evaluate the effect of the types of polymer and composition of mixed fibrous multilayered meshes on MC3T3-E1 cell differentiation. On comparing the color intensity for these scaffolds, it is clear that the osteogenic level of cell differentiation in microfibrous meshes of PLGA (Fig. 13a) was minimum in comparison to that of the mixed fibrous meshes of PLGA-Col (Fig. 13b) and PLGA-Col-HA (Fig. 13c). The results of Alizarin Red staining of mixed fibrous meshes of PLGA and collagen are in complete agreements with the trends obtained for cells proliferation by MTT assay on these scaffolds. In comparison to microfibrous meshes of PLGA, the mixed fibrous meshes of PLGA-Col have shown a slight increase in osteogenesis of MC3T3-E1 cells (Fig. 13b) but the degree of osteogenesis has increased significantly on mixed fibrous of PLGA-Col-HA (Fig. 13c) [73]. These findings have clearly indicated that multilayered scaffolds of mixed fibrous meshes of PLGA-Col were more osteogenic and the addition of nHA has further enhanced their osteogenetic properties due to the presence of calcium for mineralization [58, 59]. The results of Alizarin Red staining have clearly indicated that the osteogenetic properties of the scaffolds was influenced significantly by the structures and composition of multilayered scaffolds fabricated by dual extrusion electrospinning technique.

### von Kossa Assay

**Fig. 14:**
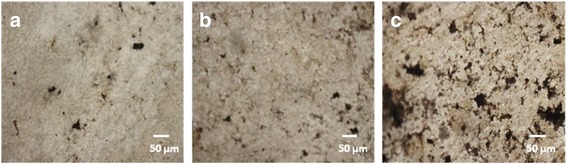
von Kossa assay for MC3T3-E1 cells cultured for 15 days on micro fibrous PLGA scaffolds **(a)**, micro/nano mixed fibrous multilayered scaffolds of PLGA and Col **(b)**, and PLGA and Col-HA **(c)**

The expression of non-collagenous osteogenic matrix proteins such as osteonectin (OSN), OSP, and OCN is a diagnostic marker for the post-proliferative mineralization stage of osteoblasts. However, detectable expression of these proteins takes place at a later stage of osteogenesis [74]. The regulation and control of osteocalcin protein at post-proliferative stage of osteoblasts are controlled by osteocalcin mRNA, which could be detected at early stage using reverse transcription polymerase chain reaction (RT-PCR) [75] or using advanced DNA microarray method [76]. The DNA microarray method is found to be more sensitive in comparison to labor-intensive semiquantitative RT-PCR method, which gives data for a few gene transcripts. Since detection of mRNA markers for the expression of OSN, OSP, and OCN proteins is time consuming and costly in comparison to simplified von Kossa protocol for detection of OCN protein marker by mineralization; hence, von Kossa protocol has been used for assaying the effect of architectural structures of 3D scaffolds on cell differentiation and mineralization as reported by earlier workers [77]. To complement the results obtained by Alizarin Red staining for osteogenesis of MC3T3-E1 cells on fabricated mixed fibrous multilayered scaffolds of collagen and PLGA, the assaying of calcium released during osteogenesis of MC3T3-E1 cells has also been carried out by exchanging ultraviolet radiation-reduced calcium with silver ions as per von Kossa protocol. The assaying of calcium mineralization by von Kossa has been carried out after seeding MC3T3-E1 cells for 15 days as shown in Fig. 14.

On comparing the black spots of deposited silver ions on microfibrous scaffolds of PLGA (Fig. 14a) and micro/nano mixed fibrous multilayered scaffolds of PLGA and Col (Fig. 14b), it is apparent that micro/nano mixed fibrous multilayered scaffolds of PLGA and Col-HA were more osteoconducting for differentiation and mineralization of MC3T3-E1 cells. Micro/nano mixed fibrous multilayered scaffolds of PLGA and Col-HA (Fig. 14c) were able to produce well-developed black spots that confirmed high osteogenic properties of PLGA and Col-HA due to the presence of nHA in the mixed fibrous scaffolds. The von Kossa assaying of scaffolds for cell differentiation and mineralization is found to be in complete agreement with the trends shown by Alizarin Red staining of cells. This has indicated that the presence of collagen in nHA mixed fibrous scaffolds has contributed significantly toward mineralization of MC3T3-E1 cells.

### Actin Cytoskeleton Assay

**Fig. 15:**
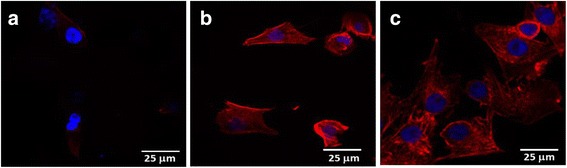
Actin cytoskeleton images of MC3T3-E1 cells cultured for 3 days on microfibrous PLGA scaffolds **(a)**, micro/nano mixed fibrous multilayered scaffolds of PLGA and Col **(b)**, and PLGA and Col-HA **(c)**

Cytoskeletal filamentous actin is a dynamic network of associated proteins found in all eukaryotic cells to mediate a variety of essential biological functions, including intra- and extracellular movement and also provide structural supports [78]. The quantifiable differentiation in orientation and distribution of actin filaments takes place with the progression of cell cycle. The progression of cytoskeleton actin also depends on the interactions of extracellular materials [34]. The local adhesion and F-actin filament expression in the cells were determined by fluorescent staining with TRITC. The emitted rhodamine-phalloidin fluorescence intensity (Ex 488 nm, Em 590) was used to infer the expression of F-actin in MC3T3-E1 cells (Fig. 15). The images of about 50 cells were acquired in triplicates at each time of point to get an average view of F-actin expression using Nikon-Elements AR 3.0 software package. The counter staining with DAPI was used to stain the nucleus to investigate the proliferation of MC3T3-E1 cells on fabricated scaffolds of different composition and architectures. The fluorescent images of MC3T3-E1 cells cultured on different scaffolds have shown significant variations in the organization of F-actin and number of cell nucleus (Fig. 15).

The polygonal morphology of MC3T3-E1 cells has shown a significant variation on microfibrous scaffolds of PLGA (Fig. 15a) and micro/nano mixed fibrous scaffolds of PLGA and Col and PLGA and Col-HA (Fig. 15b, c). The F-actin expression of microfibrous scaffolds of PLGA is found to be negligible (Fig. 15a) in comparison to micro/nano mixed fibrous multilayered PLGA and Col scaffolds with PLGA and Col-HA scaffolds (Fig. 15b, c).

The expression of F-actin in the micro/nano mixed fibrous multilayered scaffolds of PLGA and Col-HA is found to be more prominent (Fig. 15c) in comparison to the micro/nano mixed fibrous multilayered scaffolds of PLGA and Col (Fig. 15b). This result has indicated that the actin cytoskeleton of the cells cultured for 3 days on micro/nano mixed fibrous scaffold of PLGA and Col was more expressed due to the presence of collagen and was further expressed on using scaffolds having nHA (PLGA and Col-HA).

## Conclusions

Electrospinning is a simple and potentially useful technique for the fabrication of micro/nano fibrous scaffolds using solutions of various biomaterials. In this study, dual extrusion electrospinning technique was found to be a novel approach in controlling the architectural structures and composition of 3D scaffolds by controlling the electrospinning and solution parameters. The multilayered scaffolds with alternate arrangements of microfibrous PLGA meshes with micro/nano mixed fibrous meshes of PLGA and collagen have been fabricated successfully using dual extrusion electrospinning technique. The fabricated scaffolds were characterized using FT-IR and X-ray photoelectron spectroscopy for confirming the presence of collagen and nHA. The bioactivity of fabricated scaffolds has been evaluated as a function of collagen density and the presence of nHA in the scaffolds. As the result, the presence of nanofibrous collagen and hydroxyapatite nanorods has contributed significantly in controlling the surface area and bioactivity of the scaffolds such as the adhesion, proliferation, and differentiation of MC3T3-E1 cells. The dual extrusion electrospinning technique would be used further for designing 3D scaffolds with different topologies and compositions for drug delivery and bone tissue engineering in our ongoing programs for the applications of biomaterials in the fields of biomedical research.
